# Predictors of diagnostic transition from major depressive disorder to bipolar disorder: a retrospective observational network study

**DOI:** 10.1038/s41398-021-01760-6

**Published:** 2021-12-20

**Authors:** Anastasiya Nestsiarovich, Jenna M. Reps, Michael E. Matheny, Scott L. DuVall, Kristine E. Lynch, Maura Beaton, Xinzhuo Jiang, Matthew Spotnitz, Stephen R. Pfohl, Nigam H. Shah, Carmen Olga Torre, Christian G. Reich, Dong Yun Lee, Sang Joon Son, Seng Chan You, Rae Woong Park, Patrick B. Ryan, Christophe G. Lambert

**Affiliations:** 1 University of New Mexico Health Sciences Center, Department of Internal Medicine, Center for Global Health, Albuquerque, NM USA; 2 Janssen Research and Development, Raritan, NJ USA; 3 Vanderbilt University, Department of Biomedical Informatics, Department of Medicine, Department of Biostatistics, Nashville, TN USA; 4 Tennessee Valley Healthcare System VA, Nashville, TN USA; 5 Veterans Affairs Informatics and Computing Infrastructure, VA Salt Lake City Health Care System, Salt Lake City, UT USA; 6 University of Utah, Department of Internal Medicine, Salt Lake City, UT USA; 7 Columbia University Irving Medical Center, Department of Biomedical Informatics, New York, NY USA; 8 Stanford University, Stanford Center for Biomedical Informatics Research, Stanford, CA USA; 9 IQVIA, Real World Solutions, Brighton, UK; 10 IQVIA, Real World Solutions, Cambridge, MA USA; 11 Ajou University School of Medicine, Department of Psychiatry, Suwon, Republic of Korea; 12 Ajou University School of Medicine, Department of Biomedical Informatics, Suwon, Republic of Korea; 13 University of New Mexico Health Sciences Center, Department of Internal Medicine, Center for Global Health, Division of Translational Informatics, Albuquerque, NM USA

**Keywords:** Depression, Bipolar disorder

## Abstract

Many patients with bipolar disorder (BD) are initially misdiagnosed with major depressive disorder (MDD) and are treated with antidepressants, whose potential iatrogenic effects are widely discussed. It is unknown whether MDD is a comorbidity of BD or its earlier stage, and no consensus exists on individual conversion predictors, delaying BD’s timely recognition and treatment. We aimed to build a predictive model of MDD to BD conversion and to validate it across a multi-national network of patient databases using the standardization afforded by the Observational Medical Outcomes Partnership (OMOP) common data model. Five “training” US databases were retrospectively analyzed: IBM MarketScan CCAE, MDCR, MDCD, Optum EHR, and Optum Claims. Cyclops regularized logistic regression models were developed on one-year MDD-BD conversion with all standard covariates from the HADES PatientLevelPrediction package. Time-to-conversion Kaplan-Meier analysis was performed up to a decade after MDD, stratified by model-estimated risk. External validation of the final prediction model was performed across 9 patient record databases within the Observational Health Data Sciences and Informatics (OHDSI) network internationally. The model’s area under the curve (AUC) varied 0.633–0.745 (*µ* = 0.689) across the five US training databases. Nine variables predicted one-year MDD-BD transition. Factors that increased risk were: younger age, severe depression, psychosis, anxiety, substance misuse, self-harm thoughts/actions, and prior mental disorder. AUCs of the validation datasets ranged 0.570–0.785 (*µ* = 0.664). An assessment algorithm was built for MDD to BD conversion that allows distinguishing as much as 100-fold risk differences among patients and validates well across multiple international data sources.

## Introduction

Mood disorders are one of the three leading causes of disability worldwide [1], with major depressive disorder (MDD) affecting more than 17 million Americans [2] and bipolar disorder (BD) affecting 7 million Americans annually [3]. Both MDD and BD are chronic debilitating psychiatric conditions, with overlapping neurobiology and symptoms (recurrent depressive episodes), however, BD is diagnosed if at least one manic/hypomanic episode was present during the patient lifetime [4]. It is debatable whether MDD represents an earlier stage of BD or is part of the same illness [5–7], given high MDD to BD prospective conversion rates (20-year rate of 25% [8] and annual rate of 0.8–3.9% [9, 10]). A retrospective study following up subjects with MDD for 8 years showed that 7.6–12.1% of them subsequently had their MDD diagnosis changed to BD, with a mean time to change being 1.89–2.98 years [11]. The nosological distinction between BD and MDD is of great clinical significance since patients with BD have less favorable illness course and outcomes [12] and require different treatments. For example, antidepressants might trigger a manic mood switch among those with BD [13]. It is suggested that the use of antidepressants by patients with unrecognized BD can contribute to their drug resistance, making them difficult to treat [11].

A recent meta-analysis failed to identify factors that were consistently confirmed to predict MDD to BD conversion across studies [10]. Among the reported predictors of diagnosis conversion were younger patient age, treatment resistance, early depression onset, higher depression severity, multiple depressive episodes, family history of mood disorders, co-existing alcohol/substance abuse, attention-deficit/hyperactivity disorder, anxiety disorders, psychoses, suicide attempts, personality disorders, hospitalization, as well as rapid mood cycling, psychotherapy, living alone, prior use of psychotropic drugs, and others [8, 14–18]. Contradicting data are reported across the literature on the role of sex, age, depression onset, and severity in MDD-BD diagnostic transition.

This work set out to develop a predictive model of the conversion from MDD to BD, and to validate the model across a multi-national network of patient databases using the standardization afforded by the Observational Medical Outcomes Partnership (OMOP) common data model. The final goal was to develop a simple clinically useful risk assessment algorithm to help practitioners to recognize BD as early as possible among the patients presenting with MDD, and thus, to shorten the duration of untreated illness and mitigate the iatrogeny.

## Patients and methods

This was a retrospective observational cohort study to develop a prognostic model of MDD diagnostic conversion to BD within a one-year period. Patient data on diagnoses, observations, provider visits, procedures performed, and medications filled were extracted from our local OMOP data repository and analyzed within our computational health platform [19]. Five “training” US databases were used to develop the model: IBM MarketScan Commercial Claims and Encounters Database (CCAE, 2001–2018), IBM MarketScan Medicare Supplemental Database (MDCR, 2001–2018), IBM MarketScan Multi-State Medicaid Database (MDCD, 2005–2018), Optum de-identified Electronic Health Record Dataset (Optum EHR, 2005–2018), and Optum de-Identified Clinformatics Data Mart Database (Optum claims, 2001–2018). The sociodemographic and clinical data were extracted on all individuals who had a first observable diagnosis of MDD and then either had subsequent BD diagnosis within one year or not. Patient inclusion criteria were: age >10 years at the time of the first recorded MDD diagnosis (“index visit”); at least one year of observation before the index visit; no diagnosis of BD, schizophrenia, or schizoaffective disorder at any time point before the index visit (from the start of patient database coverage), and no antipsychotic, antidepressant, lithium, or mood-stabilizing anticonvulsant (MSA) use recorded in the given database at any time point before the index visit. A one-year observation period before the index visit was required to collect the relevant covariates on each patient to predict their future diagnostic transition. All selected individuals with MDD were followed up to 1 year, but if BD diagnosis occurred within this one-year period, the observation was stopped on the day that BD diagnosis was coded. Thus, patients with MDD-BD diagnosis conversion within one year were considered as “cases”, and those who did not convert during one year after the index visit were considered as a “control group”.

Using the Cyclops R package [9], data-driven regularized logistic regression models were developed on one-year MDD-BD conversion with all standard covariates from the PatientLevelPrediction R library [10] of the Observational Health Data Sciences and Informatics (OHDSI). The covariates included patient age, sex, month of the index visit, diagnoses, drug exposures, and procedures. There were tens of thousands of covariates included in our initial model, and we ran multiple rounds of simplification to get hundreds of them, and, eventually, just several covariates in our final model. Binary measures were used to label the presence or absence of a covariate. Age was binned into 5-year intervals, except for the 60–69 interval, which was used as a “reference range” covering both the Medicare database (with only individuals 65 + y.o.) and CCAE (with only individuals under 65 y.o.) to avoid collinearity and enable comparison of our five training datasets with a common reference age.

The modeling was performed for each of the five training datasets and included two stages: regression with the original tens of thousands of database covariates, and subsequent regression/simplification with a reduced set of covariates, based on subject matter-guided selection and grouping.

In the first stage of the modeling process, which was purely data-driven, for covariates classified as “conditions” in the database, we included the “parent” (or “ancestor”) codes from the SNOMED vocabulary for the whole group of disorders (e.g. “Disorder of cardiovascular system” or “Disorder of endocrine system”) to capture multiple codes covered by these umbrella terms. Similarly, “drug” covariates were grouped by the drug ingredient which could include varying brands, dosages, and release mechanisms of the same pharmacological agent. Cyclops regression was used which has a procedure employing regularized regression with cross-validation, which drops covariates that do not add to the model’s discriminative performance, and thus, reduces the pool of thousands of candidate predictors into several hundred variables. We also employed XGBoost modeling as an alternative to logistic regression to determine if better performance could be achieved by modeling interactions and nonlinear relationships. We described the model’s performance (i.e. its capacity to robustly discriminate between cases and controls) by reporting the receiver operator characteristic area under the curve (AUC) across five training datasets.

The second stage of the modeling process was data and human-driven. A clinical subject matter expert re-inspected the already reduced set of predictors and grouped them into “composite” covariates related to the same medical problem (value 1 was used to code “present” and 0—“absent”). For example, a composite variable “substance misuse” would have a value of 1 if the patient had either alcohol abuse or opioid dependence in the year before the index visit. Individual components of such composite covariates were required to have the same direction and magnitude of effect across the five training datasets. Covariates with inconsistent directionality across the datasets, as well as those with hazard ratio values close to one (thus, not clinically significant), were excluded from the analysis. Computational definitions for each “composite” covariate were then expanded to incorporate additional descendent terms using SNOMED and drug ingredient vocabulary hierarchies, and logistic regression models were built using these covariates. We then retrained the model on each database using Cyclops with the composite covariates (see our electronic data source https://github.com/ohdsi-studies/BipolarMisclassificationValidation/tree/master/inst/cohorts for composite covariate definitions). For each composite covariate, an average coefficient was calculated across models for the five databases. These values were then multiplied by 10 and rounded to the nearest integer to enable an easily computable overall risk score per person by summing the rounded values across the final set of composite covariates.

The final regression model was then shared with our collaborators across the US and internationally for external validation. We also performed a sensitivity analysis on feature importance using our five training databases among new cases of MDD that appeared in 2019 and onwards (after model development), by examining the impact on AUC of including/excluding each variable in the model.

To explore how consistent the long-term risk of diagnostic transition was for each patient as a function of an estimated individual’s short-term (one year) risk, we performed secondary Kaplan–Meier survival analysis on the time from MDD to BD diagnosis conversion (with censoring) as a function of prediction model risk score, going forward into the future as far as 10 years from the index visit. Also, for each training and validation dataset, we generated a Cox regression model of time to MDD conversion (with right censoring) as a function of a smoothing spline of the per-person risk score, with a score of zero as reference. This way we could estimate how much the overall risk score was associated with risk of MDD-BD transition.

For each training dataset, we have also built a “calibration plot” which uses locally estimated scatterplot smoothing (LOESS) to plot the actual/observed risk as a function of the predicted risk [20]. A model is considered well-calibrated when the predicted risks match the observed risks.

External validation of the final prediction model was performed using several validation datasets within the OHDSI network including Columbia University (CUIMC), Ajou University in South Korea (AUSOM) [13], STAnford medicine Research data Repository (STARR) [21], IQVIA (including Germany, France, Belgium, and US records), Japan Medical Data Center database (JMDC) [11], as well as the US Veterans Health Administration EMR. This validation approach was similar to the one used in a recent OHDSI study [12].

The present study was approved by the institutional review boards (IRBs) of the respective collaborating institutions, where applicable. The Ajou University Hospital IRB number is AJIRB-MED-MDB-20-034. Approval for the use of STARR is provided by the Stanford Institutional Review Board, protocol 53248. The use of IBM and Optum databases was reviewed by the New England IRB and was determined to be exempt from IRB approval.

**Fig. 1:**
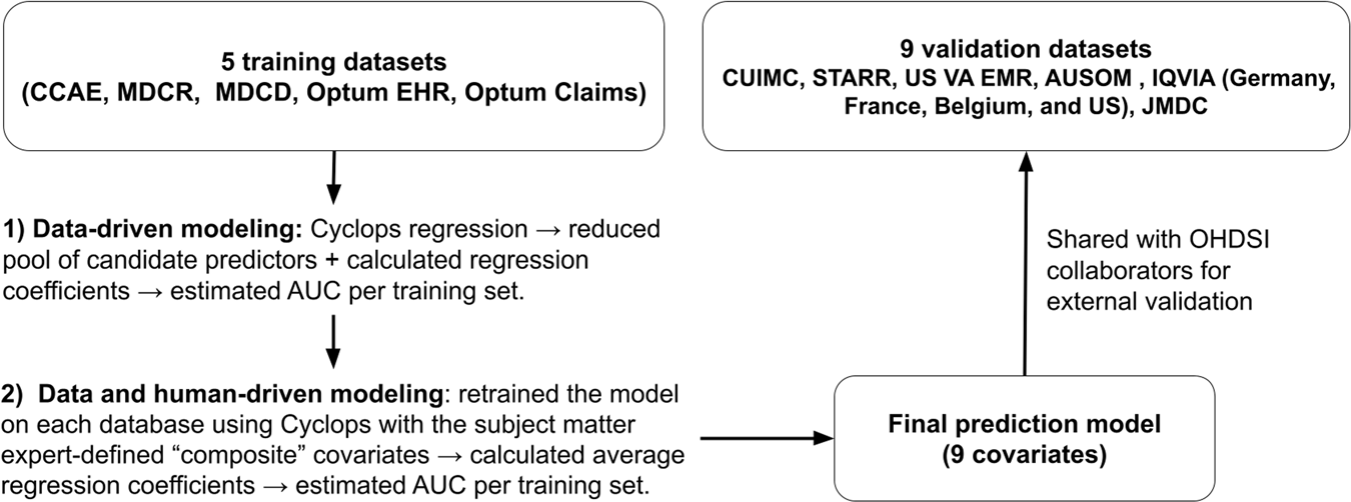
The overall schema of the methods used to create and disseminate the model of one-year prediction of major depressive disorder to bipolar disorder. See abbreviations in the main text.

The overall schema of the methods used is displayed in Fig. 1.

## Results

There were a total of 2,687,578 patients included in all five training databases meeting the study eligibility criteria. Patient characteristics are reported in Supplementary Table S1 as the average fractions of MDD to BD conversions for each of the model covariates per training database. Experimental employment of XGBoost versus Cyclops logistic regression did not result in higher AUC values, therefore all further models used Cyclops to develop parsimonious models. Also, no appreciable reduction in AUC was observed during the simplification of the model when moving from hundreds of covariates with Cyclops towards our final model. As a result of the human-modified/corrected data analysis, our final simple additive score model identified nine variables predicting one-year MDD transition to BD, where the age variable is represented by 11 groups. Each of the variables was assigned a score (positive score means a higher risk of diagnostic transition, and negative score means a lower risk of diagnostic transition): age at MDD onset 10–14 (+11); age 15–29 (+12); age 30–34 (+10); age 35–39 (+9); age 40–44 (+8); age 45–49 (+7); age 50–54 (+5); age 55–59 (+3); age 60–69 (+0, a reference group); age 70–74 (−3); age over 75 (−5); mild MDD at index visit (−5); severe MDD at index visit (+5); psychosis at MDD index visit (+10); other mental disorders one year before but excluding the index visit (+2); anxiety disorders and/or use of anti-anxiety drugs one year before and including the index MDD visit (+1); pregnancy one year before and during the index visit (−3); substance misuse one year before and during the index visit (+5); self-harm thoughts or actions one year before and during the index MDD visit (+9). Note that the covariate “patient sex” was dropped from the model at the final stage of analysis since it was not conferring sufficient risk for diagnosis transition (coefficient was close to zero).

Table 1 shows the prediction performance of the model on the five training datasets. The training model AUCs varied 0.633-0.745 with an average of 0.689 across the databases.

**Table 1:** Performance of the final score-based model predicting diagnostic transition from major depressive disorder (MDD) to bipolar disorder (BD) within one year, in “training” US datasets.

Data Source	AUC (95% CI)	Sample size (met inclusion criteria)	Outcome count	Outcome %
**MDCR**	0.632 (0.608–0.656)	48,979	609	1.243
**MDCD**	0.662 (0.658–0.666)	253,811	15,789	6.221
**CCAE**	0.690 (0.687–0.693)	677,233	24,325	3.592
**Optum EHR**	0.714 (0.712–0.717)	1,187,111	26,070	2.196
**Optum claims**	0.745 (0.742–0.748)	520,444	14,301	2.748

*MDCR* IBM MarketScan Medicare Supplemental Database, *MDCD* IBM MarketScan Multi-State Medicaid Database, *CCAE* IBM MarketScan Commercial Claims and Encounters Database, *Optum EHR* Optum De-identified Electronic Health Record Dataset, *Optum claims* Optum De-Identified Clinformatics Data Mart Database, *AUC* area under the curve, *CI* confidence interval.

**Fig. 2:**
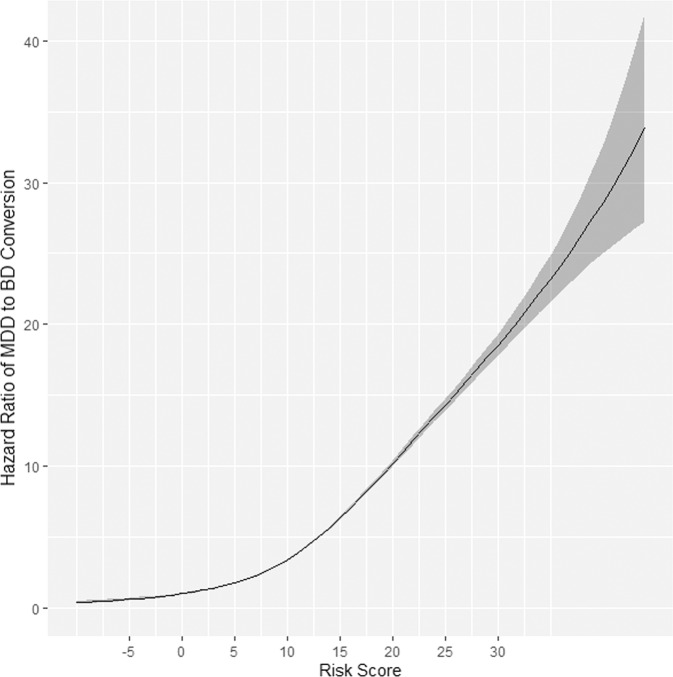
The hazard ratio of diagnostic MDD to BD conversion as a function of risk score in the CCAE database. The gray “shadow” indicates the respective 95% confidence interval for the hazard ratio (*y-*axis). A risk score of 0 was used as a reference. MDD—major depressive disorder. BD - bipolar disorder. CCAE—IBM MarketScan Commercial Claims and Encounters database.

**Fig. 3:**
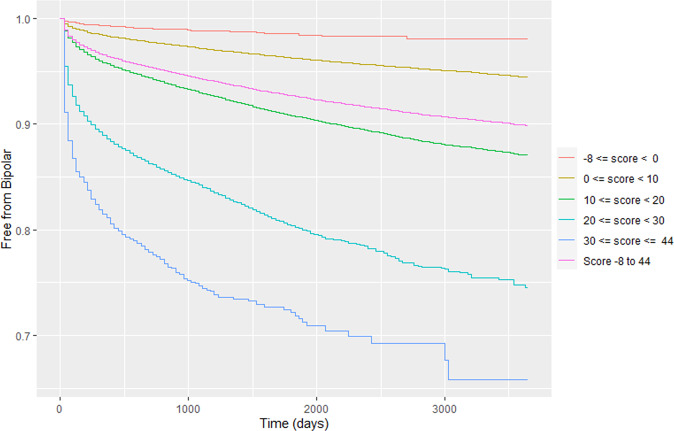
Kaplan–Meier curves of diagnostic conversion in the CCAE dataset based on risk score range. Each colored line represents a different score range (from top to the bottom): orange: -8 to −1; yellow: 0–9; green: 10–19; teal: 20–29; dark blue: 30–44, bright pink: any score. CCAE—IBM MarketScan Commercial Claims and Encounters database. MDD—major depressive disorder. BD—bipolar disorder.

Figure 2 shows the >100-fold hazard ratio range for MDD-BD transition over the range of covariate risk scores in the CCAE database. Thus, in the CCAE database, for a patient with zero risk score, the hazard ratio of diagnostic transition would be one, and for a patient with a risk score of 25, it would be 15. Therefore, the latter patient would have a 15-times higher relative risk of transitioning from MDD to BD, compared to the former patient. Supplementary Figs. S1–S14 contain risk score-hazard ratio plots for other databases, which show a similar magnitude of effect for the overall risk score per patient. Supplementary Figs. S15–S19 contain training models’ calibration plots which show an overall monotonically increasing relationship between model-predicted risk and the observed transition fraction. The MDCR model underestimated the conversion risk since the smoothed actual proportion of conversions was consistently higher than predicted. Figure 3 shows the Kaplan–Meier survival curves broken out by risk score range for the CCAE database.

Supplementary Figs. S20–S28 contain graphs with Kaplan–Meier survival curves for all training and most validation datasets (some collaboration sites did not provide their graphs to maximally protect patient confidentiality).

**Fig. 4:**
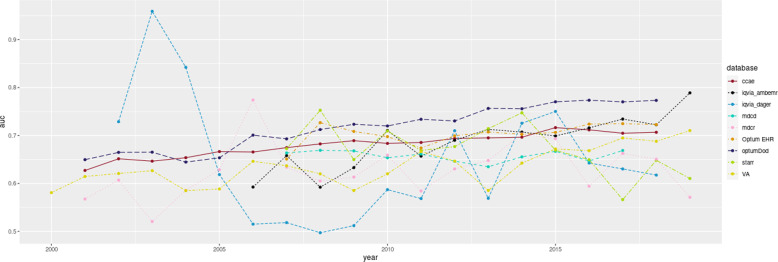
Performance of predictive model for one-year MDD-BD diagnosis conversion depending on a data recording year. AUC—area under the curve. CCAE—IBM MarketScan Commercial Claims and Encounters Database (US), IQVIA_ambemr—IQVIA Ambulatory database for US, IQVIA_DAGER—IQVIA database for Germany, MDCD—IBM MarketScan Multi-State Medicaid Database, MDCR—IBM MarketScan Medicare Supplemental Database, Optum EHR—Optum de-identified electronic health record dataset, Optum claims—Optum De-Identified Clinformatics Data Mart Database, STARR—STAnford medicine Research data Repository, VA—US Veterans Administration database.

**Fig. 5:**
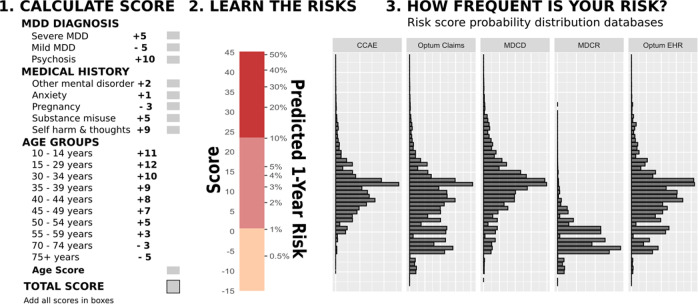
The risk assessment algorithm for a one-year diagnostic transition from MDD (major depressive disorder) to BD (bipolar disorder). CCAE—IBM MarketScan Commercial Claims and Encounters Database, MDCR—IBM MarketScan Medicare Supplemental Database, MDCD—IBM MarketScan Multi-State Medicaid Database, Optum EHR—Optum De-identified Electronic Health Record Dataset, Optum claims—Optum De-Identified Clinformatics Data Mart Database. “MDD diagnosis” scores are for depression severity and the presence of psychotic features within the index episode. “Medical history” events could occur at any time prior to and including the index visit.

External validation of our final predictive model showed performance better than random prediction (AUC > 0.50) across all the validation databases (Table 2), with AUCs ranging 0.570–0.785 (average 0.664). Figure 4 demonstrates the model’s performance in different databases (training and validation) as a function of time. As a result of our integrative data analysis from different databases, we developed a simple, clinically meaningful algorithm to estimate the individual patient’s risk of MDD diagnosis transition to BD within one year after the index visit (Fig. 5). Interactive results are available for viewing within an R Shiny application at https://data.ohdsi.org/MDDinBipolar/.

**Table 2:** Performance of the final score-based model predicting diagnostic transition from major depressive disorder (MDD) to bipolar disorder (BD) within one year, in “validation” (US and international) datasets.

Data source	AUC (95% CI)	N of patients who met inclusion criteria	N of patients with BD outcome in 1 year	Proportion of MDD patient population with BD outcome
CUIMC (US EHR)	0.570 (0.543–0.598)	5611	457	8.145
JMDC (Japanese Claims)	0.610 (0.545–0.675)	1303	67	5.142
IQVIA DAFR (French EMR)	0.615 (0.482–0.749)	1910	17	0.890
IQVIA DAGER (German EMR)	0.628 (0.597–0.658)	127,353	315	0.247
STARR (US EHR)	0.646 (0.613–0.678)	27,266	290	1.064
Veterans Health Administration (US EMR)	0.670 (0.665–0.675)	359,449	9246	2.570
IQVIA Ambulatory (US EMR)	0.691 (0.680–0.702)	148,343	1548	1.044
IQVIA Belgium (Belgian EMR)	0.757 (0.602–0.912)	667	7	1.050
AUSOM (South Korean EMR)	0.785 (0.724–0.847)	2570	30	1.167

*AUC* area under the curve, *N* number, *BD* bipolar disorder, *MDD* major depressive disorder, *EHR* electronic health records, *EMR* electronic medical records, *CUIMC* Columbia University database (US), *JMDC* Japan Medical Data Center database, *DAFR* IQVIA data from France, *DAGER* IQVIA data from Germany, *STARR* STAnford medicine Research data Repository (US), *AUSOM* Ajou University data from South Korea.

Supplementary Table S2 shows the results of our covariate sensitivity analysis when the final prediction model was applied prospectively on our training databases for patients with MDD onset in 2019. The full model’s AUC and the AUC when each of the 9 variables was excluded are shown (age was excluded with all of its 5-year interval variables). Excluding a covariate typically resulted in lower AUC values or the same AUC values as in the full model, though only age was statistically significant within the larger Optum and CCAE databases.

## Discussion

In our final regression model, younger patient age, higher severity of the initial depressive episode, the presence of psychotic features during the index depression, and anxiety were predictive of MDD diagnostic transition to BD within one year.

The association of diagnostic transition with a younger age can have the following explanation: BD typically manifests at a younger age, which is supported by previous studies aiming to distinguish MDD from BD [22, 23]. The association of mood episode severity with BD transition is consistent with other findings that the highest severity depressive episodes occur in BD-I, followed by BD-II and MDD [12]. This observation is also supported by results from the study of Goldberg et al., where bipolar converters were found to have more severe depressive symptoms or psychotic symptoms than non-converters [24], as well as by results of other prospective and retrospective studies [23–25]. Anxiety (as evidenced by an anxiety disorder diagnosis or the intake of anti-anxiety medication) was among the MDD-BD switching predictors in our study. A recent cross-sectional study has shown that the prevalence of comorbid anxiety disorders was significantly higher in patients with BD (53.2%) than in patients with MDD (37.2%) [26]. Another study found that comorbid anxiety in patients with mood disorders can serve as a marker of clinical severity [27].

Older age, mild baseline depression, and pregnancy were found to be predictors of a lower MDD-BD transition risk within one year in our study. The pregnancy association may be explained by (1) there is generally a high level of vigilance among healthcare providers regarding recognition of postpartum depression, thus, hypomania/mania symptoms can remain unrecognized in this category of patients, (2) pregnant/postpartum women might enroll disproportionately higher with obstetric services, than with psychiatric ones, even if they have already established mental healthcare, (3) pregnancy/delivery itself, pharmacological treatment of pregnancy-associated conditions, or pharmacotherapy of MDD with a selected set of non-teratogenic antidepressants during the perinatal period could hypothetically have a protective effect on mania/hypomania symptoms.

The clinical utility of our study results is in informing scientific discussions about nosological differences between BD and MDD and helping to recognize BD manifesting with depression relatively early. The latter remains a major diagnostic challenge in clinical psychiatry. Patient surveys found that one-third of BD respondents waited for more than ten years before receiving the correct diagnosis despite actively seeking help [28, 29], and saw an average of four physicians [29]. In its turn, lack of timely treatment of BD due to misdiagnosis can lead to an increased risk of suicide, long-lasting functional impairment, unemployment, family and legal issues, frequent hospitalizations, and higher healthcare costs than with appropriate BD treatment [30–33], and early switch to lithium or other mood-stabilizing agents in high-risk patients might be warranted [34]. Considering the identified predictors (early onset of depression, presence of severe depression, and psychotic features) might help to promote vigilance among clinicians regarding the possibility of underlying BD diagnosis in depressed patients and encourage them to ask clarifying questions about manic/hypomanic episodes.

Assigning each patient an overall “risk score” based on our proposed risk assessment algorithm might be a useful clinical tool. For example, to calculate the risk of one-year BD conversion in a 21-year old (+12 points) female initially diagnosed with mild MDD (−5 points), with a history of an anxiety disorder (+1 point) and substance abuse (+5 points) but with no other risk factors, you would use Fig. 5 part 1 to calculate her score of 13 (12-5 + 1 + 5) and then use part 2 to convert the score into a predicted risk of ~4%.

As shown in Fig. 2, the risk of diagnostic conversion from MDD to BD can differ ~10-fold between a patient with zero risk score and a patient with a risk score of 20. The difference between the “worst” case scenario when a patient has all the “unfavorable” factors for transition (score = +44, corresponding to a 50% conversion rate) versus the “best” scenario when a patient has only “favorable” factors (score = −10 in a 75+-year-old person presenting with mild MDD and negligible probability of pregnancy at this age, corresponding to a < 0.5% conversion rate) gives us a > 100-fold range of risk (Fig. 5).

Our predictive model was successfully validated in several external datasets from different countries, which supports its potential applicability across different healthcare systems. However, its overall performance was modest (average 0.69 in training datasets, and 0.66 in validation datasets), and was influenced by individual database cohort characteristics and recording practices. The small sample sizes from some of the external validation sites (Belgium, France, Japan, South Korea) led to wide confidence intervals in the performance estimates. The simple score model performed worse in CUIMC, but this database appears to have a much higher outcome rate, so this may indicate a different type of patient population or perhaps some differences in data recording. The relatively better performance of the AUSOM database may be explained by chance and the relatively small population who fit the inclusion criteria. The German and French data had lower percentages of one-year diagnostic conversion, which may be due to the data being extracted from primary care datasets, not covering enough psychiatric services. The predictive model was quite stable over time (from year to year), except for the IQVIA DAGER database, which represents German data, and had a relatively small number of outcomes (315 spread over 17 years) that could have led to the wide variance in yearly risk estimates.

Our prospective covariate sensitivity analyses on the patients with MDD onset in 2019 from the training databases suggest that the model performance and covariate directionality are stable moving forward in time, though only the age covariate had a significant effect on model performance when one variable was considered at a time.

On average over all 14 databases, the one-year conversion rate from MDD to BD was 2.666%. While this number may appear relatively low, if it was to be sustained for 10 years, a conversion rate of 1-(1–0.0266)^10^ = 23.6% might be expected in a decade. While our Kaplan–Meier curves suggest these rates tend to drop off after the first few years, we do observe that persons who fall on the high end of risk in our models can exceed a 1 in 3 chance of conversion within a decade, representing an important population to screen for BD diagnosis and treatment.

*Study limitations*. Our data were extracted from electronic health records and administrative claims data, which have known limitations including incomplete data recording, variations of diagnostic decision criteria used by providers and of granularity/amount of patient-reported information during each visit. The data were unavailable before the patient database enrollment or the database start date. The external validity of the model could be better overall, some validation datasets had comparable AUCs to those of the training sets, and some were too small to accurately assess. Because of the limitations of the health insurance claims data, factors such as laboratory test results were not included in this study. We acknowledge that there was the potential for overfitting in the development of the models, but this was mitigated by validation in external databases with comparable models’ performance. We also recognize that it was not necessarily the first episode of MDD/BD in a patient’s life captured in our data (some patients started observation at 65 years old). Since hypomanic symptoms are often not reported by a patient and are not accounted for when making a diagnosis, our database could miss a portion of BD type II cases.

## Conclusions

Our approach produced a simple, clinically understandable model for predicting one-year risk of diagnosis conversion from MDD to BD that validates well across multiple international data sources. Early onset of MDD, presence of psychotic features, severe depression, substance misuse, and suicidality can serve as clinical predictors of prospective transition of a patient to BD diagnostic group and should get close attention from clinicians. Despite moderate AUC performance, our model can identify patients spanning a 100-fold magnitude difference in risk of MDD to BD transition. Accounting for interactions and nonlinear relationships between the variables using XGBoost did not result in higher AUC values, suggesting that substantial improvements in model performance are unlikely to be gained by more feature engineering of the training databases, though temporal relationships might be explored further. Because one can code and bill for MDD without specifying severity, we anticipate that models that go beyond claims records to incorporate physician notes and results of depression psychometric assessment may lead to more precise predictions of the risk of future conversion.

## Supplementary information

Supplemental Material
