# Supplementary material for: Predictors of diagnostic transition from major depressive disorder to bipolar disorder: a retrospective observational network study
Source: Transl Psychiatry. 2021 Dec 20;11:642. doi: 10.1038/s41398-021-01760-6 (PMC8688463; doi:10.1038/s41398-021-01760-6)

**Supplementary materials**

**Predictors of diagnostic transition from major depressive disorder to bipolar disorder: a retrospective observational network study**

Anastasiya Nestsiarovich, MD, PhD^1^; Jenna M. Reps, PhD^2^; Michael E. Matheny, MD, MS, MPH^3,4^; Scott L. DuVall, PhD^5,6^; Kristine E. Lynch, PhD^5,6^; Maura Beaton, MS^7^; Xinzhuo Jiang, MS^7^; Matthew Spotnitz, MD, MPH^7^; Stephen R. Pfohl, BS^8^; Nigham H. Shah, MBBS, PhD^8^; Carmen Olga Torre, MS^9^; Christian G. Reich, MD, PhD^10^; Dong Yun Lee, MD^11^; Sang Joon Son, MD, PhD^11^; Seng Chan You, MD, MS^12^; Rae Woong Park, MD, PhD^12^; Patrick B. Ryan, PhD^2,7^; Christophe G. Lambert, PhD^1,13*^

^1^University of New Mexico Health Sciences Center, Department of Internal Medicine, Center for Global Health, Albuquerque, NM, USA

^2^Janssen Research and Development, Raritan, NJ, USA

^3^Vanderbilt University, Department of Biomedical Informatics, Department of Medicine, Department of Biostatistics, Nashville, TN, USA

^4^ Tennessee Valley Healthcare System VA, Nashville, TN, USA

^5^ Veterans Affairs Informatics and Computing Infrastructure, VA Salt Lake City Health Care System, Salt Lake City, UT, USA

^6^ University of Utah, Department of Internal Medicine, Salt Lake City, UT, USA

^7^ Columbia University Irving Medical Center, Department of Biomedical Informatics, New York, NY, USA

^8^Stanford University, Stanford Center for Biomedical Informatics Research, Stanford, CA, USA

^9^IQVIA, Real World Solutions, Brighton, UK

^10^IQVIA, Real World Solutions, Cambridge, MA, USA

^11^Ajou University School of Medicine, Department of Psychiatry, Suwon, Republic of Korea

^12^Ajou University School of Medicine, Department of Biomedical Informatics, Suwon, Republic of Korea

^13^University of New Mexico Health Sciences Center, Department of Internal Medicine, Center for Global Health, Division of Translational Informatics, Albuquerque, New Mexico, USA.

**Table S1. Characteristics of the patients included (those who had diagnosis transition from MDD to BD and those who had not).**

**Table S2. Prospective sensitivity analysis of model covariates on training datasets for patients with MDD onset in 2019.**

**Figure S1. The hazard ratio of diagnostic conversion from MDD to BD as a function of an individual patient’s risk score in CCAE (IBM MarketScan Commercial Claims and Encounters database).**

**Figure S2. The hazard ratio of diagnostic conversion from MDD to BD as a function of an individual patient’s risk score in MDCD (IBM MarketScan Multi-State Medicaid Database).**

**Figure S3. The hazard ratio of diagnostic conversion from MDD to BD as a function of an individual patient’s risk score in MDCR (IBM MarketScan Medicare Supplemental Database).**

**Figure S4. The hazard ratio of diagnostic conversion from MDD to BD as a function of an individual patient’s risk score in Optum EHR (Optum de-identified electronic health record) dataset.**

**Figure S5. The hazard ratio of diagnostic conversion from MDD to BD as a function of an individual patient’s risk score in Optum Claims (Optum De-Identified Clinformatics Data Mart Database).**

**Figure S6. The hazard ratio of diagnostic conversion from MDD to BD as a function of an individual patient’s risk score in Starr (STAnford medicine Research data Repository).**

**Figure S7. The hazard ratio of diagnostic conversion from MDD to BD as a function of an individual patient’s risk score in CUIMC (Columbia University data).**

**Figure S8. The hazard ratio of diagnostic conversion from MDD to BD as a function of an individual patient’s risk score in VA (US Veterans Administration database).**

**Figure S9. The hazard ratio of diagnostic conversion from MDD to BD as a function of an individual patient’s risk score in JMDC (Japan Medical Data Center database).**

**Figure S10. The hazard ratio of diagnostic conversion from MDD to BD as a function of an individual patient’s risk score in IQVIA AMBEMR (IQVIA Ambulatory database for US).**

**Figure S11. The hazard ratio of diagnostic conversion from MDD to BD as a function of an individual patient’s risk score in IQVIA DAFR (French electronic medical records).**

**Figure S12. The hazard ratio of diagnostic conversion from MDD to BD as a function of an individual patient’s risk score in IQVIA DAGER (IQVIA database for Germany).**

**Figure S13. The hazard ratio of diagnostic conversion from MDD to BD as a function of an individual patient’s risk score in IQVIA Belgium database.**

**Figure S14. The hazard ratio of diagnostic conversion from MDD to BD as a function of an individual patient’s risk score in AUSOM (Ajou University database from South Korea).**

**Figure S15. Calibration plots for diagnosis conversion from MDD to BD within one year in CCAE database (IBM MarketScan Commercial Claims and Encounters Database).**

**Figure S16. Calibration plots for diagnosis conversion from MDD to BD within one year in Optum Claims database.**

**Figure S17. Calibration plots for diagnosis conversion from MDD to BD within one year in Optum EHR database.**

**Figure S18. Calibration plots for diagnosis conversion from MDD to BD within one year in MDCD (IBM MarketScan Multi-State Medicaid Database).**

**Figure S19. Calibration plots for diagnosis conversion from MDD to BD within one year MDCR (IBM MarketScan Medicare Supplemental) Database.**

**Figure S20. Kaplan-Meier survival curves broken out by individual patient’s risk score range for CCAE database.**

**Figure S21. Kaplan-Meier survival curves broken out by individual patient’s risk score range for IQVIA Amber database.**

**Figure S22. Kaplan-Meier survival curves broken out by individual patient’s risk score range for IQVIA DAGER database (Germany).**

**Figure S23. Kaplan-Meier survival curves broken out by individual patient’s risk score range for JMDC database (Japan).**

**Figure S24. Kaplan-Meier survival curves broken out by individual patient’s risk score range for MDCD database.**

**Figure S25. Kaplan-Meier survival curves broken out by individual patient’s risk score range for MDCR database.**

**Figure S26. Kaplan-Meier survival curves broken out by individual patient’s risk score range for Optum Claims database.**

**Figure S27. Kaplan-Meier survival curves broken out by individual patient’s risk score range for STARR database.**

**Figure S28. Kaplan-Meier survival curves broken out by individual patient’s risk score range for Optum EHR database.**

**Table S1. Characteristics of the MDD patients included (those who had diagnosis transition from MDD to BD and those who had not).**

|  | **Optum Claims** | | **Optum EHR** | | **MDCD** | | **MDCR** | | **CCAE** | |
| --- | --- | --- | --- | --- | --- | --- | --- | --- | --- | --- |
| **Covariate:** | Bipolar | No Bipolar | Bipolar | No Bipolar | Bipolar | No Bipolar | Bipolar | No Bipolar | Bipolar | No Bipolar |
| **age group: 10-14** | 0.081 | 0.040 | 0.042 | 0.038 | 0.191 | 0.180 | NA | NA | 0.097 | 0.068 |
| **age group: 15-19** | 0.236 | 0.098 | 0.121 | 0.093 | 0.237 | 0.211 | NA | NA | 0.276 | 0.171 |
| **age group: 20-24** | 0.129 | 0.061 | 0.137 | 0.079 | 0.082 | 0.062 | NA | NA | 0.163 | 0.110 |
| **age group: 25-29** | 0.090 | 0.050 | 0.124 | 0.075 | 0.096 | 0.074 | NA | NA | 0.087 | 0.067 |
| **age group: 30-34** | 0.084 | 0.065 | 0.110 | 0.074 | 0.082 | 0.064 | NA | NA | 0.084 | 0.088 |
| **age group: 35-39** | 0.081 | 0.069 | 0.093 | 0.073 | 0.068 | 0.054 | NA | NA | 0.073 | 0.094 |
| **age group: 40-44** | 0.066 | 0.066 | 0.079 | 0.072 | 0.050 | 0.043 | 0.000 | 0.000 | 0.065 | 0.093 |
| **age group: 45-49** | 0.054 | 0.064 | 0.079 | 0.080 | 0.053 | 0.045 | 0.002 | 0.001 | 0.056 | 0.092 |
| **age group: 50-54** | 0.045 | 0.060 | 0.072 | 0.086 | 0.047 | 0.051 | 0.008 | 0.004 | 0.044 | 0.088 |
| **age group: 55-59** | 0.035 | 0.054 | 0.056 | 0.085 | 0.038 | 0.053 | 0.008 | 0.007 | 0.032 | 0.077 |
| **age group: 60-64** | 0.020 | 0.045 | 0.036 | 0.069 | 0.023 | 0.042 | 0.033 | 0.011 | 0.022 | 0.053 |
| **age group: 65-69** | 0.022 | 0.077 | 0.022 | 0.054 | 0.012 | 0.035 | 0.238 | 0.227 | 0.001 | 0.000 |
| **age group: 70-74** | 0.020 | 0.081 | 0.013 | 0.040 | 0.008 | 0.028 | 0.217 | 0.220 | NA | NA |
| **age group: 75-79** | 0.015 | 0.065 | 0.009 | 0.035 | 0.005 | 0.022 | 0.184 | 0.184 | NA | NA |
| **age group: 80-84** | 0.013 | 0.057 | 0.005 | 0.031 | 0.003 | 0.019 | 0.141 | 0.160 | NA | NA |
| **age group: 85-89** | 0.011 | 0.048 | 0.003 | 0.017 | 0.003 | 0.017 | 0.102 | 0.118 | NA | NA |
| **age group:  90 - 94** | NA | NA | NA | NA | NA | NA | 0.059 | 0.053 | NA | NA |
| **age group:  95 - 99** | NA | NA | NA | NA | NA | NA | 0.007 | 0.013 | NA | NA |
| **age group: 100 - 104** | NA | NA | NA | NA | NA | NA | 0.002 | 0.001 | NA | NA |
| **psychiatric disorder** | 0.228 | 0.169 | 0.166 | 0.111 | 0.386 | 0.323 | 0.278 | 0.182 | 0.212 | 0.137 |
| **pregnancy** | 0.059 | 0.060 | 0.079 | 0.068 | 0.144 | 0.131 | 0.002 | 0.004 | 0.061 | 0.080 |
| **anxiety drugs and /or anxiety disorder** | 0.449 | 0.482 | 0.622 | 0.552 | 0.531 | 0.541 | 0.545 | 0.556 | 0.445 | 0.448 |
| **mild depression** | 0.057 | 0.130 | 0.031 | 0.061 | 0.055 | 0.091 | 0.049 | 0.097 | 0.057 | 0.116 |
| **severe depression** | 0.251 | 0.105 | 0.149 | 0.058 | 0.227 | 0.113 | 0.227 | 0.120 | 0.282 | 0.148 |
| **MDD with psychosis** | 0.042 | 0.009 | 0.034 | 0.006 | 0.069 | 0.026 | 0.107 | 0.021 | 0.049 | 0.012 |
| **substance use disorder** | 0.231 | 0.152 | 0.377 | 0.198 | 0.373 | 0.286 | 0.144 | 0.102 | 0.187 | 0.101 |
| **suicidal thoughts or self-harm** | 0.120 | 0.026 | 0.212 | 0.052 | 0.190 | 0.077 | 0.051 | 0.009 | 0.131 | 0.035 |

Reported are the average fractions of MDD to BD conversions for each of the model covariates per database.EHR – electronic health records; MDCD - IBM MarketScan Multi-State Medicaid Database; MDCR - IBM MarketScan Medicare Supplemental Database; CCAE - IBM MarketScan Commercial Claims and Encounters Database. MDD - major depressive disorder. BD/Bipolar - bipolar disorder.

**Table S2. Prospective sensitivity analysis of model covariates on training datasets for patients with MDD onset in 2019.**

| Database | Optum EHR | | Optum Claims | | CCAE | | MDCD | | MDCR | |
| --- | --- | --- | --- | --- | --- | --- | --- | --- | --- | --- |
|  | AUC | 95% CI | AUC | 95%CI | AUC | 95%CI | AUC | 95%CI | AUC | 95%CI |
| Full model | 0.720 | 0.71-073 | 0.752 | 0.74-0.76 | 0.690 | 0.68-0.70 | 0.654 | 0.64-0.67 | 0.568 | 0.47-0.67 |
| Excluded age covariate | 0.671 | 0.66-0.68 | 0.651 | 0.64-0.67 | 0.640 | 0.63-0.65 | 0.637 | 0.63-0.65 | 0.597 | 0.50-0.70 |
| Excluded prior mental health  disorder | 0.721 | 0.71-0.73 | 0.748 | 0.74-0.76 | 0.688 | 0.68-0.70 | 0.655 | 0.64-0.67 | 0.567 | 0.47-0.66 |
| Excluded suicidal thoughts or self-harm | 0.694 | 0.68-0.70 | 0.742 | 0.73-0.75 | 0.685 | 0.67-0.70 | 0.642 | 0.63-0.65 | 0.554 | 0.46-0.65 |
| Excluded pregnancy | 0.721 | 0.71-0.73 | 0.751 | 0.74-0.76 | 0.688 | 0.68-0.70 | 0.658 | 0.65-0.67 | 0.567 | 0.47-0.67 |
| Excluded anxiety/using anti-anxiety drugs | 0.719 | 0.71-0.73 | 0.751 | 0.74-0.76 | 0.688 | 0.68-0.70 | 0.654 | 0.64-0.67 | 0.560 | 0.46-0.66 |
| Excluded mild depression | 0.717 | 0.71-0.73 | 0.749 | 0.74-0.76 | 0.687 | 0.68-0.70 | 0.646 | 0.63-0.66 | 0.545 | 0.44-0.65 |
| Excluded severe depression | 0.712 | 0.70 | 0.746 | 0.73-0.76 | 0.677 | 0.67-0.69 | 0.646 | 0.63-0.66 | 0.556 | 0.46-0.65 |
| Excluded psychosis | 0.716 | 0.706 | 0.749 | 0.737 | 0.687 | 0.677 | 0.648 | 0.64-0.66 | 0.565 | 0.47-0.66 |
| Excluded substance use disorder | 0.697 | 0.687 | 0.745 | 0.733 | 0.679 | 0.669 | 0.623 | 0.61-0.63 | 0.547 | 0.45-0.64 |

AUC- area under the curve, CI - confidence interval, EHR - Electronic Health Record Dataset, Optum Claims - Optum De-Identified Clinformatics Data Mart Database, CCAE - IBM MarketScan Commercial Claims and Encounters Database, MDCR - IBM MarketScan Medicare Supplemental Database, MDCD - IBM MarketScan Multi-State Medicaid Database.

**Figure S1.** **The hazard ratio of diagnostic conversion from MDD to BD as a function of an individual patient’s risk score in CCAE (IBM MarketScan Commercial Claims and Encounters) database**. The grey “shadow” indicates the 95% confidence interval for the hazard ratio (axis *y*). A risk score of 0 was used as a reference.


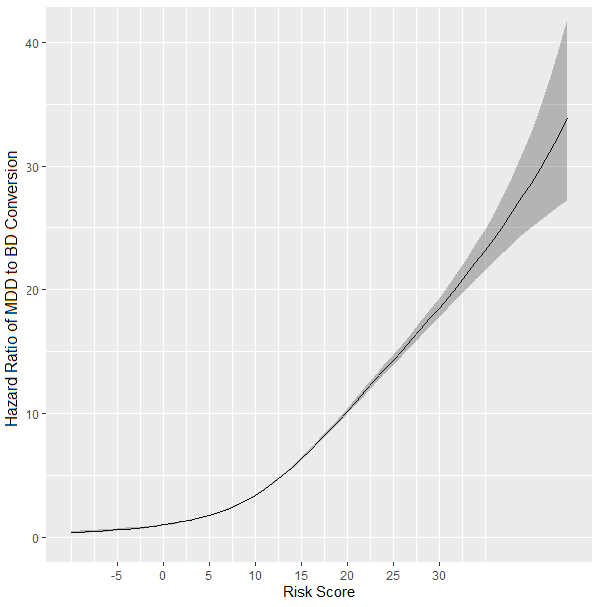


**Figure S2.** **The hazard ratio of diagnostic conversion from MDD to BD as a function of an individual patient’s risk score in MDCD (IBM MarketScan Multi-State Medicaid Database)**. The grey “shadow” indicates the 95% confidence interval for the hazard ratio (axis *y*). A risk score of 0 was used as a reference.


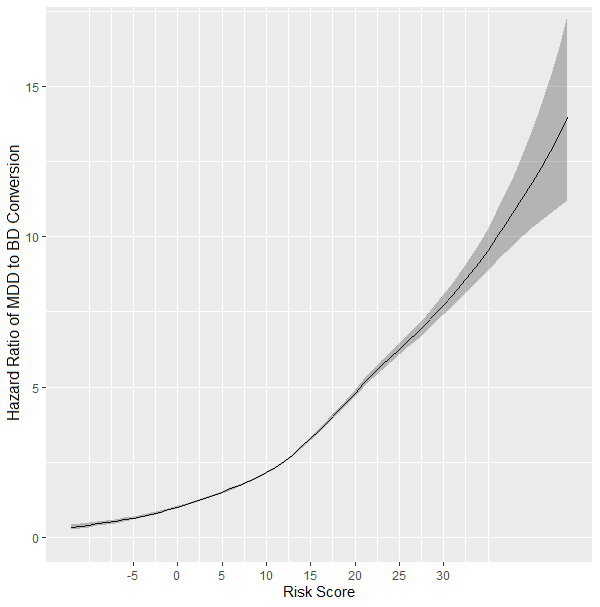


**Figure S3. The hazard ratio of diagnostic conversion from MDD to BD as a function of an individual patient’s risk score in MDCR (IBM MarketScan Medicare Supplemental Database).** The grey “shadow” indicates the 95% confidence interval for the hazard ratio (axis *y*). A risk score of 0 was used as a reference.


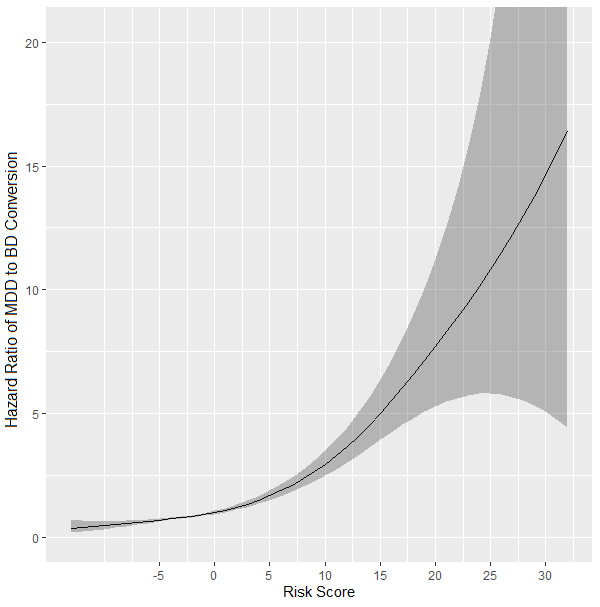


**Figure S4. The hazard ratio of diagnostic conversion from MDD to BD as a function of an individual patient’s risk score in Optum EHR (Optum de-identified electronic health record dataset)**. The grey “shadow” indicates the 95% confidence interval for the hazard ratio (axis *y*). A risk score of 0 was used as a reference.


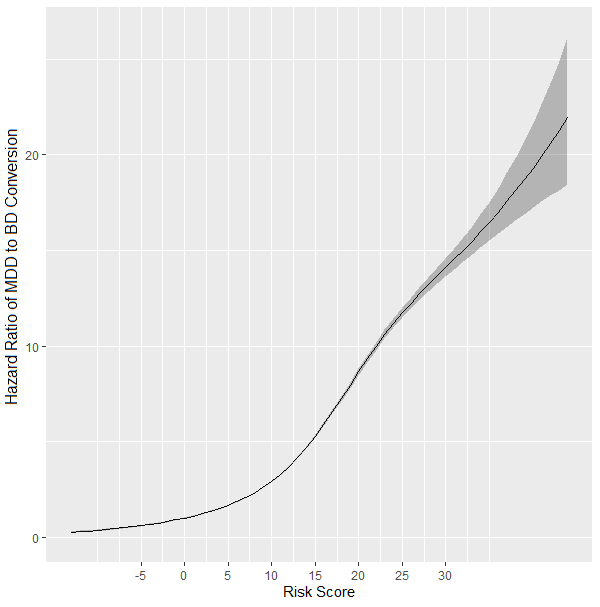


**Figure S5. The hazard ratio of diagnostic conversion from MDD to BD as a function of an individual patient’s risk score in Optum Claims (Optum De-Identified Clinformatics Data Mart Database).** The grey “shadow” indicates the 95% confidence interval for the hazard ratio (axis *y*). A risk score of 0 was used as a reference.


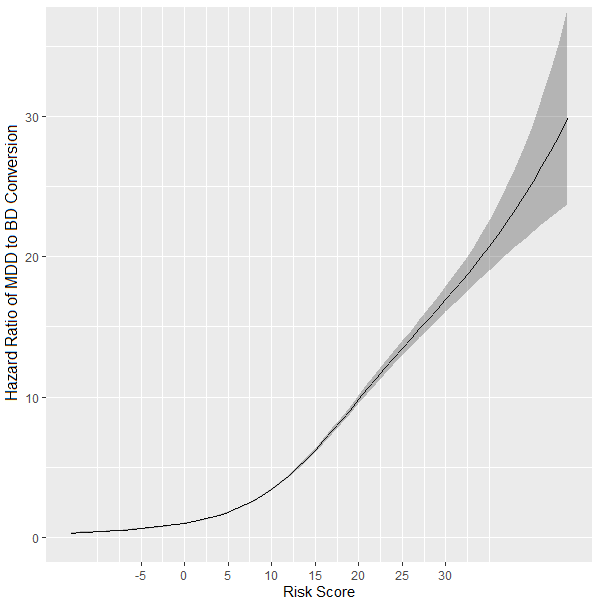


**Figure S6.** **The hazard ratio of diagnostic conversion from MDD to BD as a function of an individual patient’s risk score in Starr (STAnford medicine Research data Repository).** The grey “shadow” indicates the 95% confidence interval for the hazard ratio (axis *y*). A risk score of 0 was used as a reference.


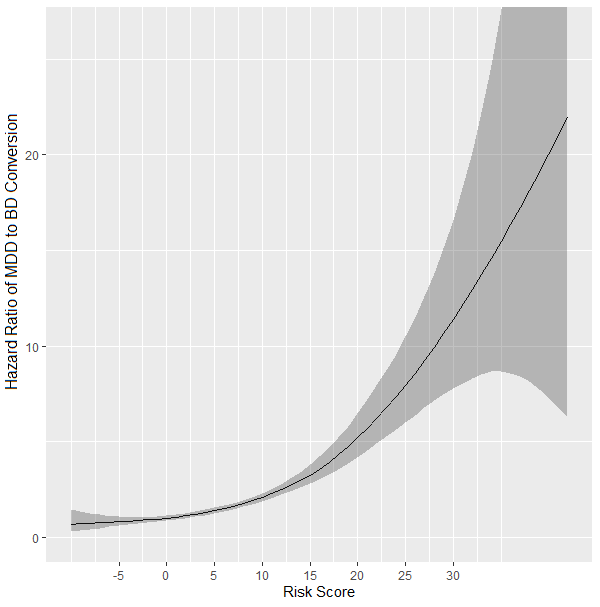


**Figure S7. The hazard ratio of diagnostic conversion from MDD to BD as a function of an individual patient’s risk score in CUIMC (Columbia University database).** The grey “shadow” indicates the 95% confidence interval for the hazard ratio (axis *y*). A risk score of 0 was used as a reference.


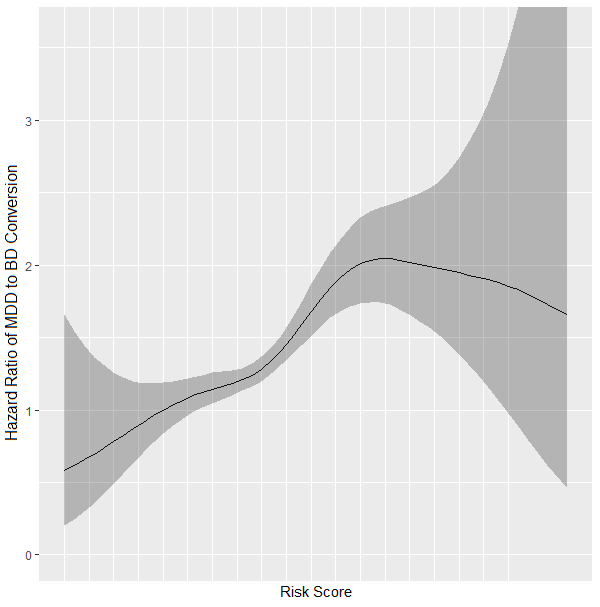


**Figure S8. The hazard ratio of diagnostic conversion from MDD to BD as a function of an individual patient’s risk score in VA (US Veterans Administration database).** The grey “shadow” indicates the 95% confidence interval for the hazard ratio (axis *y*). A risk score of 0 was used as a reference.


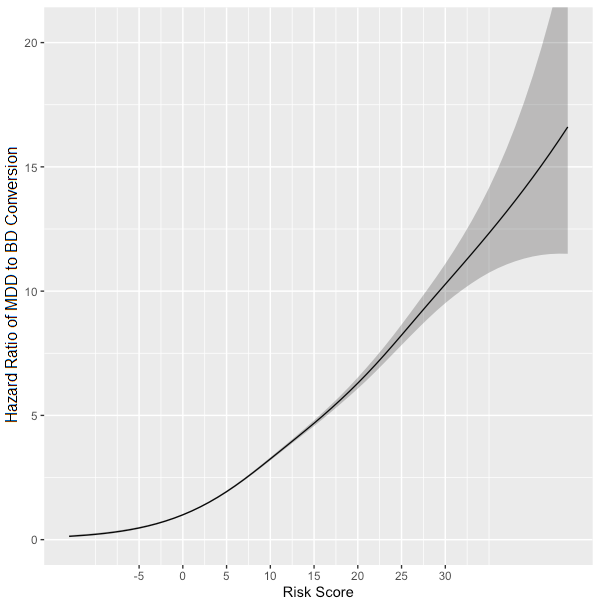


**Figure S9.** **The hazard ratio of diagnostic conversion from MDD to BD as a function of an individual patient’s risk score in JMDC (Japan Medical Data Center) database.** The grey “shadow” indicates the 95% confidence interval for the hazard ratio (axis *y*). A risk score of 0 was used as a reference.


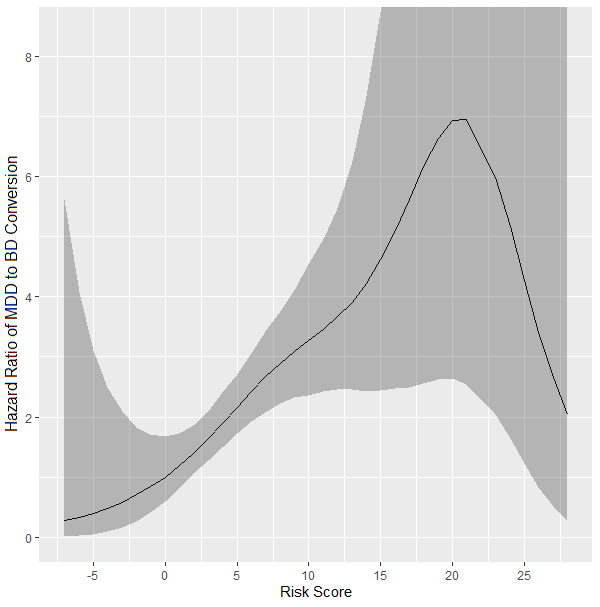


**Figure S10.** **The hazard ratio of diagnostic conversion from MDD to BD as a function of an individual patient’s risk score in IQVIA AMBEMR (IQVIA Ambulatory database for US)**. The grey “shadow” indicates the 95% confidence interval for the hazard ratio (axis *y*). A risk score of 0 was used as a reference.


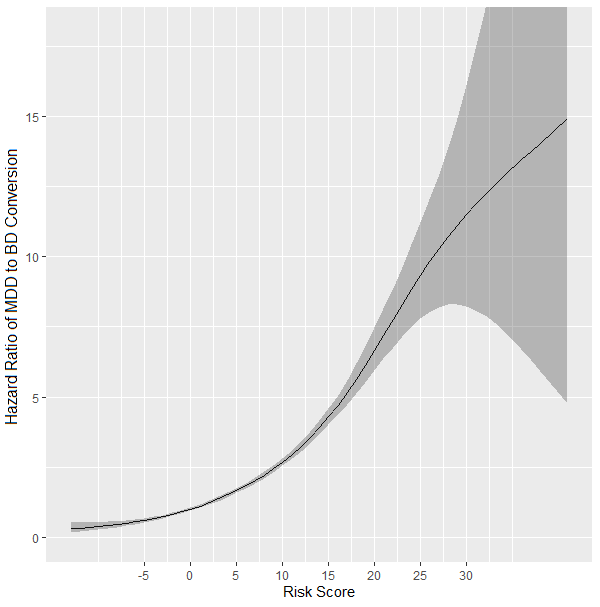


**Figure S11. The hazard ratio of diagnostic conversion from MDD to BD as a function of an individual patient’s risk score in IQVIA DAFR (French electronic medical records**). The grey “shadow” indicates the 95% confidence interval for the hazard ratio (axis *y*). A risk score of 0 was used as a reference.


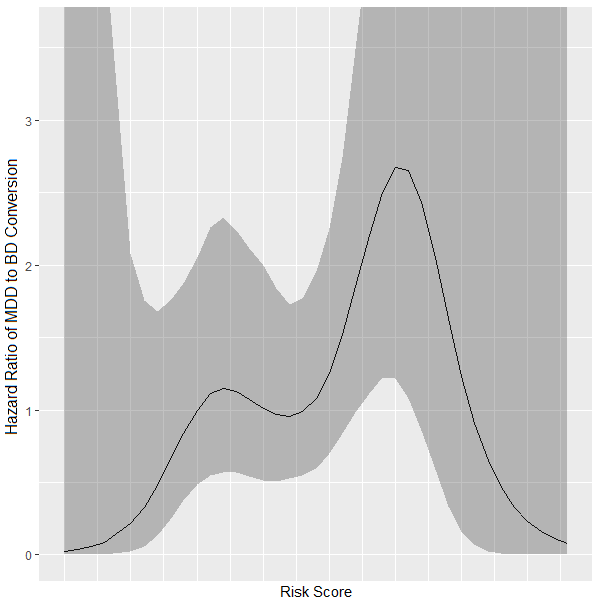


**Figure S12. The hazard ratio of diagnostic conversion from MDD to BD as a function of an individual patient’s risk score in IQVIA DAGER (IQVIA database for Germany)**. The grey “shadow” indicates the 95% confidence interval for the hazard ratio (axis *y*). A risk score of 0 was used as a reference.


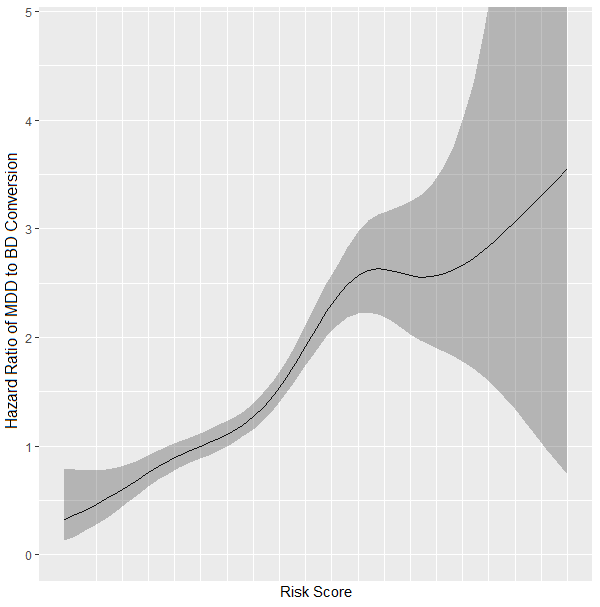


**Figure S13. The hazard ratio of diagnostic conversion from MDD to BD as a function of an individual patient’s risk score in IQVIA Belgium database**. The grey “shadow” indicates the 95% confidence interval for the hazard ratio (axis *y*). A risk score of 0 was used as a reference.


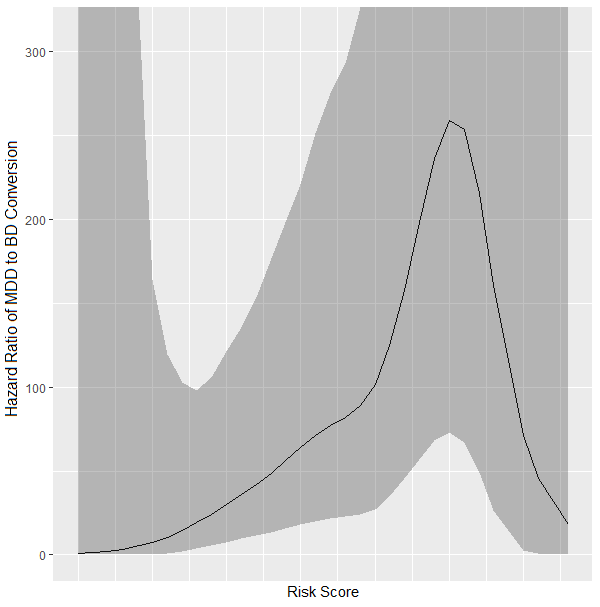


**Figure S14. The hazard ratio of diagnostic conversion from MDD to BD as a function of an individual patient’s risk score in AUSOM (Ajou University data from South Korea).** The grey “shadow” indicates the 95% confidence interval for the hazard ratio (axis *y*). A risk score of 0 was used as a reference**.**


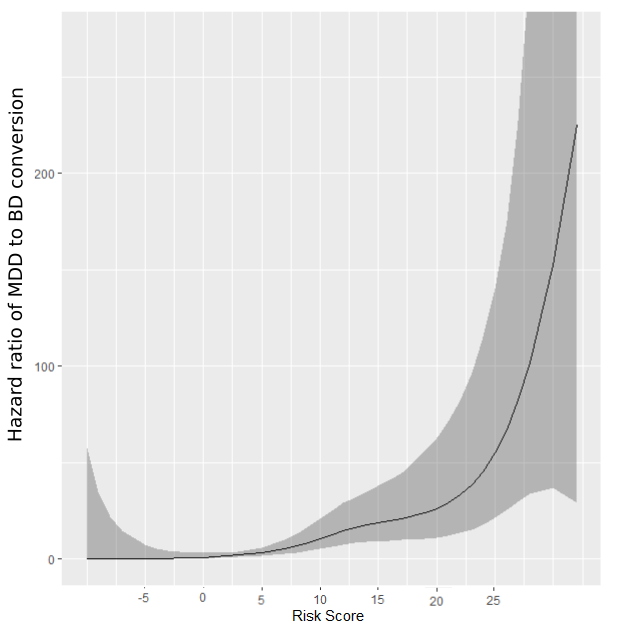


**Figure S15. Calibration plot for diagnosis conversion from MDD to BD within one year in CCAE database (IBM MarketScan Commercial Claims and Encounters Database).**

**
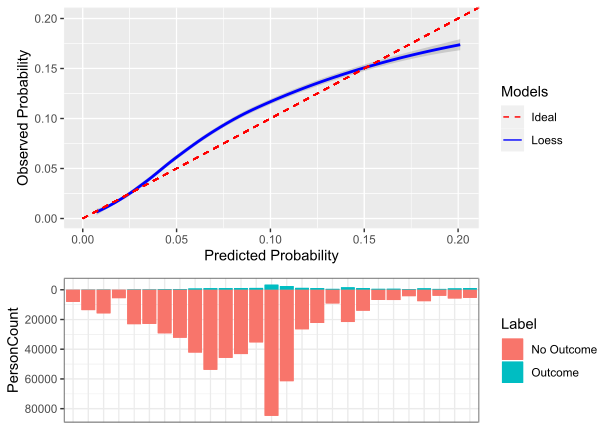
**

**Figure S16. Calibration plot for diagnosis conversion from MDD to BD within one year in Optum Claims database.**

**
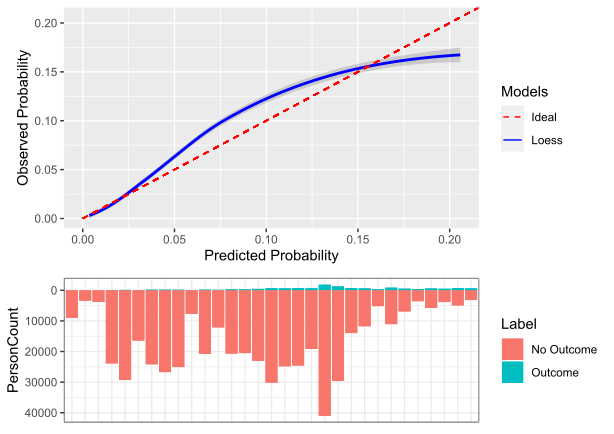
**

**Figure S17. Calibration plot for diagnosis conversion from MDD to BD within one year in Optum EHR database.**

**
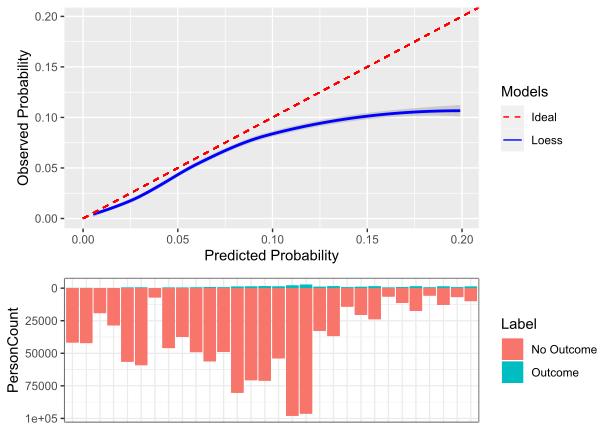
**

**Figure S18. Calibration plot for diagnosis conversion from MDD to BD within one year in MDCD database (IBM MarketScan Multi-State Medicaid Database).**

**
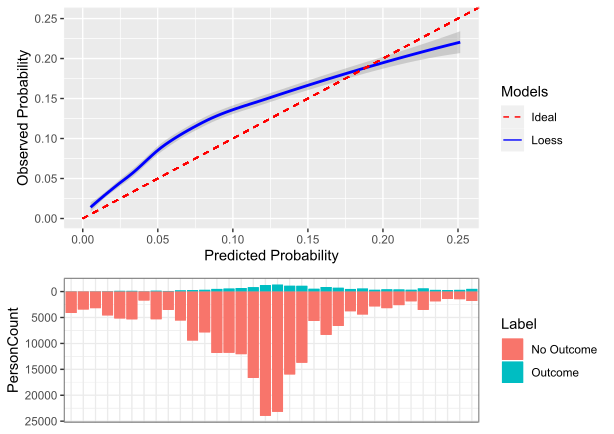
**

**Figure S19. Calibration plot for diagnosis conversion from MDD to BD within one year in MDCR database (IBM MarketScan Medicare Supplemental Database).**

**
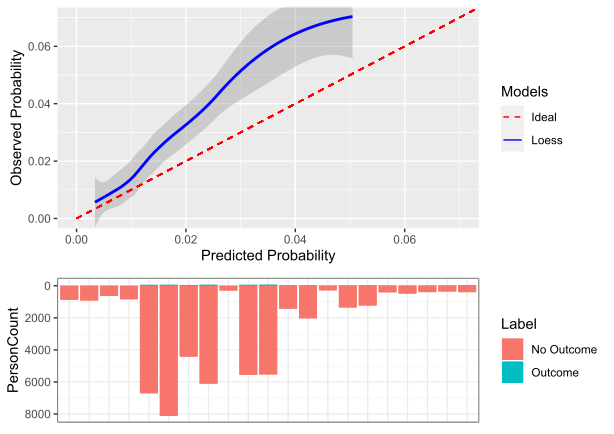
**

**Figure S20. Kaplan-Meier survival curves broken out by individual patient’s risk score range for CCAE database.** The curves demonstrate time to conversion to BD diagnosis since being first diagnosed with MDD. The “score” represents an overall risk measure assigned to each patient based on the developed prediction model of diagnostic transition.


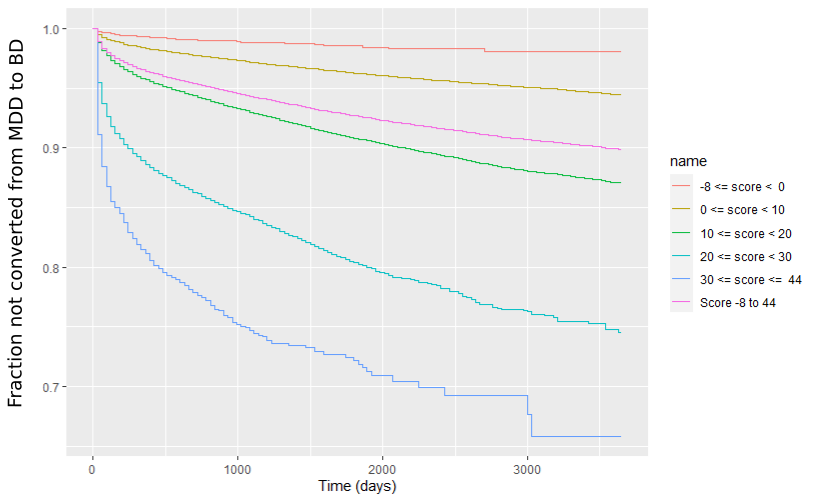


**Figure S21. Kaplan-Meier survival curves broken out by individual patient’s risk score range for IQVIA Amber database.** The curves demonstrate time to conversion to BD diagnosis since being first diagnosed with MDD. The “score” represents an overall risk measure assigned to each patient based on the developed prediction model of diagnostic transition.


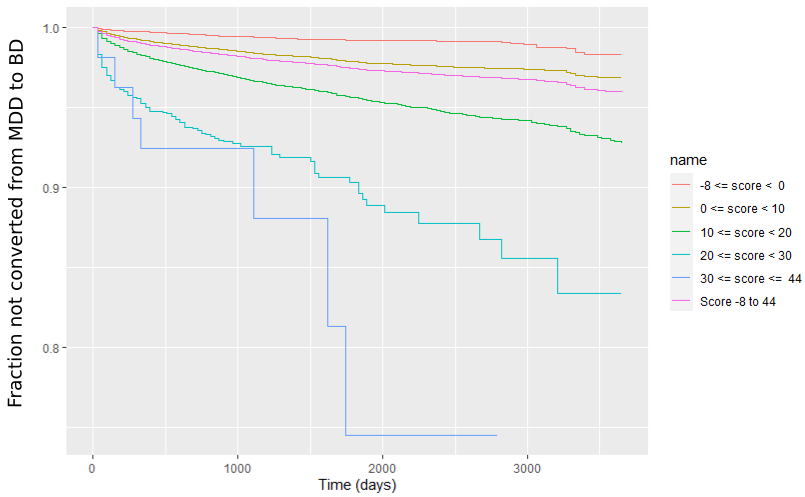


**Figure S22. Kaplan-Meier survival curves broken out by individual patient’s risk score range for IQVIA DAGER database (Germany).** The curves demonstrate time to conversion to BD diagnosis since being first diagnosed with MDD. The “score” represents an overall risk measure assigned to each patient based on the developed prediction model of diagnostic transition.


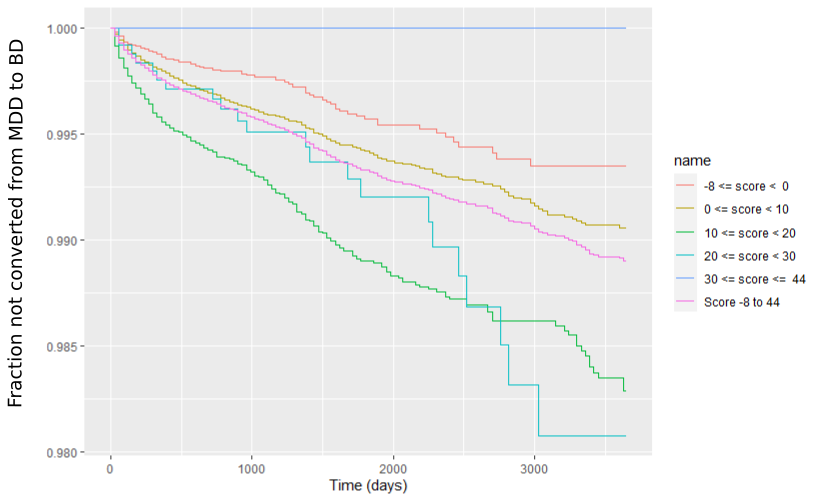


**Figure S23. Kaplan-Meier survival curves broken out by individual patient’s risk score range for JMDC database (Japan).** The curves demonstrate time to conversion to BD diagnosis since being first diagnosed with MDD. The “score” represents an overall risk measure assigned to each patient based on the developed prediction model of diagnostic transition.


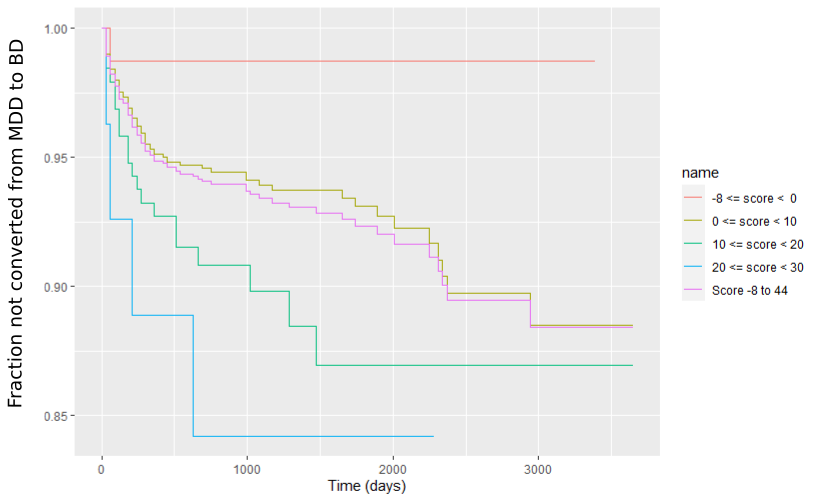


**Figure S24. Kaplan-Meier survival curves broken out by individual patient’s risk score range for MDCD database (US).** The curves demonstrate time to conversion to BD diagnosis since being first diagnosed with MDD. The “score” represents an overall risk measure assigned to each patient based on the developed prediction model of diagnostic transition.


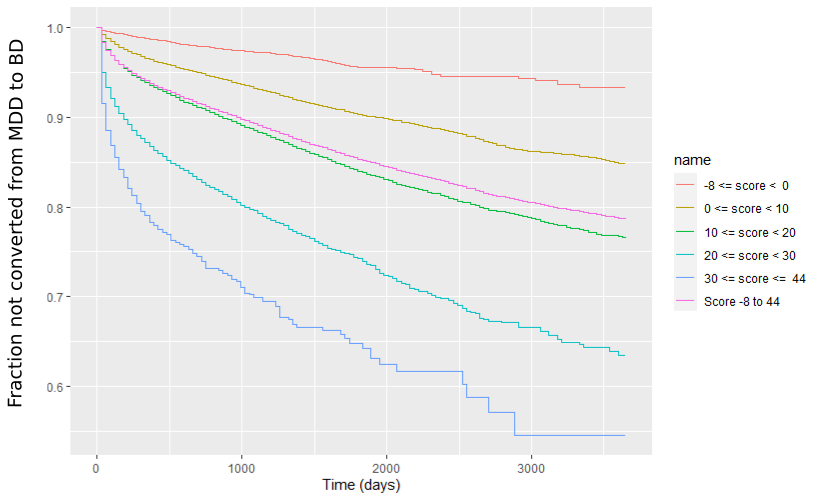


**Figure S25. Kaplan-Meier survival curves broken out by individual patient’s risk score range for MDCR database (US).** The curves demonstrate time to conversion to BD diagnosis since being first diagnosed with MDD. The “score” represents an overall risk measure assigned to each patient based on the developed prediction model of diagnostic transition.


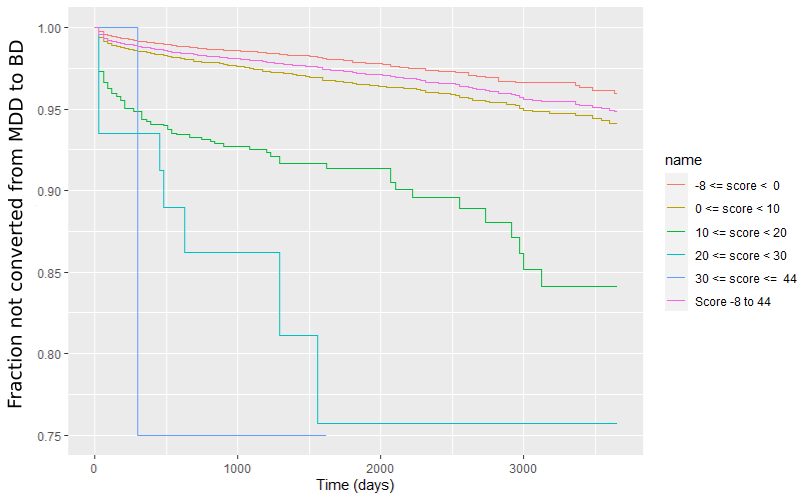


**Figure S26. Kaplan-Meier survival curves broken out by individual patient’s risk score range for Optum Claims database.** The curves demonstrate time to conversion to BD diagnosis since being first diagnosed with MDD. The “score” represents an overall risk measure assigned to each patient based on the developed prediction model of diagnostic transition.


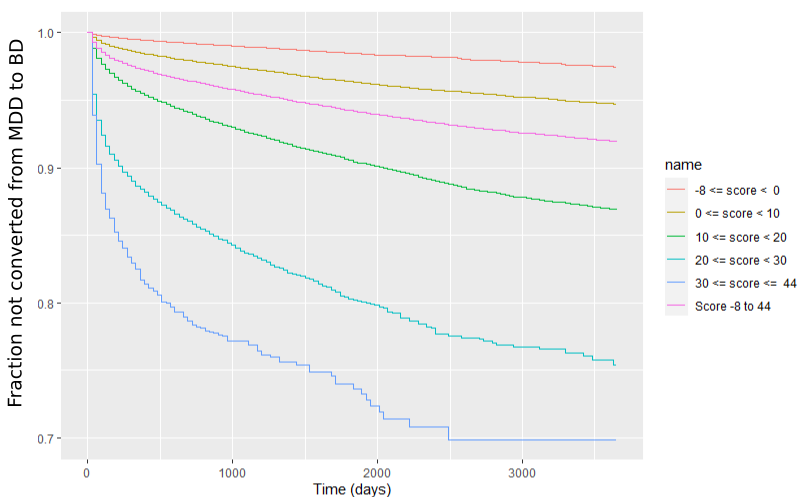


**Figure S27. Kaplan-Meier survival curves broken out by individual patient’s risk score range for STARR database.** The curves demonstrate time to conversion to BD diagnosis since being first diagnosed with MDD. The “score” represents an overall risk measure assigned to each patient based on the developed prediction model of diagnostic transition.

:


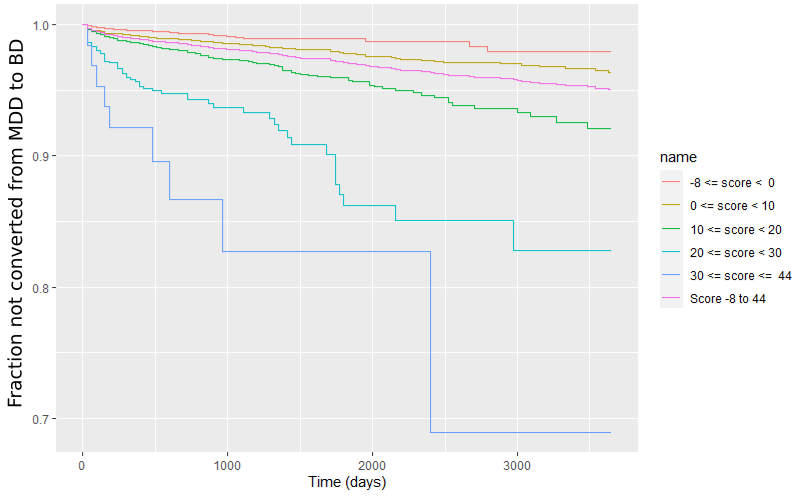


**Figure S28. Kaplan-Meier survival curves broken out by individual patient’s risk score range for Optum EHR database.** The curves demonstrate time to conversion to BD diagnosis since being first diagnosed with MDD. The “score” represents an overall risk measure assigned to each patient based on the developed prediction model of diagnostic transition.


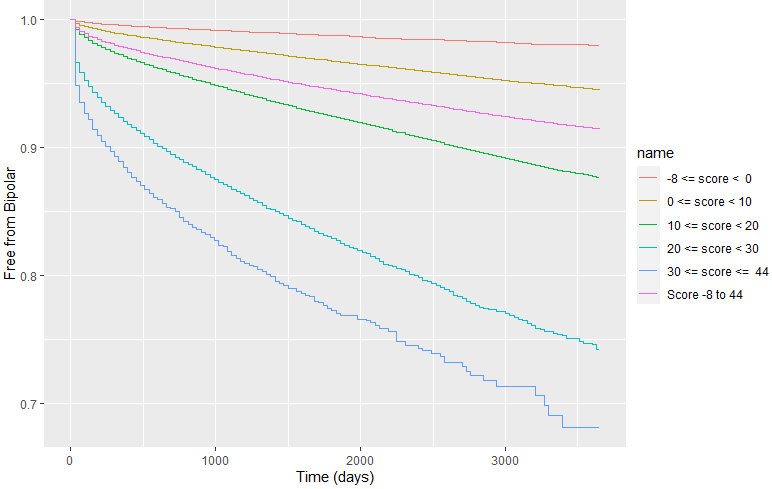

Supplement: Supplementary file 1 — Supplemental Material [file 41398_2021_1760_MOESM1_ESM.docx]
